# Silver/Graphene Oxide Nanostructured Coatings for Modulating the Microbial Susceptibility of Fixation Devices Used in Knee Surgery

**DOI:** 10.3390/ijms25010246

**Published:** 2023-12-23

**Authors:** Sorin Constantinescu, Adelina-Gabriela Niculescu, Ariana Hudiță, Valentina Grumezescu, Dragoș Rădulescu, Alexandra Cătălina Bîrcă, Stefan Andrei Irimiciuc, Oana Gherasim, Alina Maria Holban, Bianca Gălățeanu, Ovidiu Cristian Oprea, Anton Ficai, Bogdan Ștefan Vasile, Alexandru Mihai Grumezescu, Alexandra Bolocan, Radu Rădulescu

**Affiliations:** 1 Faculty of Medicine, Carol Davila University of Medicine and Pharmacy, Eroii Sanitari St. 8, 050474 Bucharest, Romania; dr.sorin.c@gmail.com (S.C.); dragos.radulescu@umfcd.ro (D.R.); alexandra.bolocan@umfcd.ro (A.B.); radu_radulescu@umfcd.ro (R.R.); 2 Research Institute of the University of Bucharest—ICUB, University of Bucharest, 050657 Bucharest, Romania; adelina.niculescu@upb.ro (A.-G.N.); alina.m.holban@bio.unibuc.ro (A.M.H.); 3 Department of Science and Engineering of Oxide Materials and Nanomaterials, University Politehnica of Bucharest, Gh. Polizu St. 1-7, 060042 Bucharest, Romania; alexandra.birca@upb.ro (A.C.B.); anton.ficai@upb.ro (A.F.); bogdan.vasile@upb.ro (B.Ș.V.); 4 Department of Biochemistry and Molecular Biology, University of Bucharest, 050095 Bucharest, Romania; bianca.galateanu@bio.unibuc.ro; 5 Lasers Department, National Institute for Lasers, Plasma and Radiation Physics, 409 Atomistilor St., 077125 Magurele, Romania; valentina.grumezescu@inflpr.ro (V.G.); stefan.irimiciuc@inflpr.ro (S.A.I.); oana.gherasim@inflpr.ro (O.G.); 6 Microbiology and Immunology Department, Faculty of Biology, University of Bucharest, 1-3 Portocalelor Lane, District 5, 77206 Bucharest, Romania; 7 Department of Inorganic Chemistry, Physical Chemistry and Electrochemistry, University Politehnica of Bucharest, 1-7 Polizu St., 011061 Bucharest, Romania; ovidiu73@yahoo.com; 8 Academy of Romanian Scientists, Spl. Independenței 54, 50085 Bucharest, Romania

**Keywords:** silver nanoparticles, graphene oxide nanosheets, MAPLE coatings, biocompatible and antimicrobial surfaces

## Abstract

Exploring silver-based and carbon-based nanomaterials’ excellent intrinsic antipathogenic effects represents an attractive alternative for fabricating anti-infective formulations. Using chemical synthesis protocols, stearate-conjugated silver (Ag@C_18_) nanoparticles and graphene oxide nanosheets (nGOs) were herein obtained and investigated in terms of composition and microstructure. Scanning electron microscopy (SEM) and transmission electron microscopy (TEM) characterizations revealed the formation of nanomaterials with desirable physical properties, while X-ray diffraction (XRD) analyses confirmed the high purity of synthesized nanomaterials. Further, laser-processed Ag@C_18_-nGO coatings were developed, optimized, and evaluated in terms of biological and microbiological outcomes. The highly biocompatible Ag@C_18_-nGO nanostructured coatings proved suitable candidates for the local modulation of biofilm-associated periprosthetic infections.

## 1. Introduction

The knee joint represents a large and complex component of the musculoskeletal system and consists of bones covered by articular cartilage and an extensive network of ligaments and muscles that provide static and dynamic stability, respectively. Knee ligaments are essential structural and functional components that synergistically contribute to the joint’s mechanical stability (by properly balancing the external and internal forces within the joint) and motion (flexion-extension and internal–external rotation) [1,2]. Mechanical injuries of knee ligaments commonly occur in highly active individuals and generally range from mild (uncomplicated stretched ligaments) to severe conditions (partially or completely ruptured ligaments accompanied by local hemorrhage, serious to extreme pain, and partial or total functional impairment) [3,4].

In severely injured knee ligaments, the surgical reconstruction of impaired tendons with autologous grafts is a preferred strategy [5,6], but polymeric constructs have also been validated as viable alternatives for the guided regeneration of ligaments [7,8]. In either case, the clinical success of knee ligament reconstruction requires proper graft positioning and solid bone fixation, achieved by using direct or indirect fixation devices. Combined with conventional sutures, wires, and staples, interference screws and flexible or adaptable fixation systems represent common fixation devices in knee ligament reconstruction [9,10]. Among these, impressive clinical outcomes have been reported when using interference devices, as they provide optimal intra-tunnel immobilization of the graft. In terms of improved joint biomechanics, comparable performances have been evidenced for bioinert (titanium-based and non-degradable thermoplastic-based) [11,12], biodegradable (magnesium-based and bioglass-based) [13,14], and bioabsorbable (biopolyester-based and biopolyester/calcium phosphate-based) [15,16] interference devices. Also, common side effects have been reported following the long-term use of such devices, including joint effusion, intra-articular screw migration, screw fragmentation or rupture, local inflammation, and infection [17,18].

Like most implantable medical devices, interference fixation devices are prone to microbial contamination and colonization [19,20], leading to the occurrence of biofilm-associated infections which cause moderate-to-severe complications that, ultimately, may lead to ligament reconstruction failure [21]. Biofilms are multicellular, surface-associated microbe communities surrounded by self-produced extracellular polymeric substances (EPSs) that provide protection for bacterial cells—thus rendering them resistant to conventional biocide/biostatic treatments—and the host’s immune response [22,23,24,25,26]. The conventional approach to reducing the frequency and severity of such infections is postoperative systemic antibiotherapy. Nonetheless, the thick EPS layer hinders the diffusion of antibiotics to biofilm-embedded cells; thus, the systemic administration of high drug doses is required. This practice may cause serious side effects, organ toxicity, low patient compliance, and the development of antibiotic-resistant bacteria [26,27,28,29,30,31].

Hospital-acquired infections are recurrent problems mainly identified in intensive care facilities and surgical and medical wards that considerably affect the patient’s quality of life, contribute to significant morbidity and mortality, and pose a tremendous financial burden on patients and healthcare systems worldwide [22,23,31,32,33,34,35]. As biofilm-related infections are very difficult to eradicate, increasing attention has started to be directed toward finding improved prevention and treatment strategies. In this context, the use of nanosized and nanostructured formulations that can prevent microbial adhesion or destroy pathogens after their attachment is of particular interest [30,36,37,38]. Specifically, nanomaterials with intrinsic biocide/biostatic effects represent auspicious alternatives to better fight pathogenic microorganisms [39].

To overcome the limitations of post-implantation systemic antibiotherapy, the surface modification of implantable devices with nanocoatings that exert local antimicrobial effects has emerged as an efficient strategy for preventing or limiting their opportunistic contamination and colonization as well as decreasing the severity of related infective complications. The laser-assisted processing of coatings represents an attractive and successful strategy for improving the surface of implantable devices [40,41]. Among the available methods, the matrix-assisted pulsed laser evaporation (MAPLE) technique has attracted tremendous interest in engineering antimicrobial coatings, being particularly well-suited for organic thin film deposition [42,43,44].

Boosting the biomechanics and healing rate of knee ligaments using fixation devices improved with bioactive coatings represents an attractive and unexplored approach to modulating their infection-free integration. Therefore, this study proposes the MAPLE processing of coatings based on representative zero-dimensional (silver nanoparticles, Ag@C_18_) and bidimensional (graphene oxide) nanomaterials and their evaluation as biocompatible interfaces in the local modulation of biofilm-associated implant infections.

## 2. Results

### 2.1. Physicochemical Investigation of Ag@C_18_ nanoparticles

**Figure 1:**
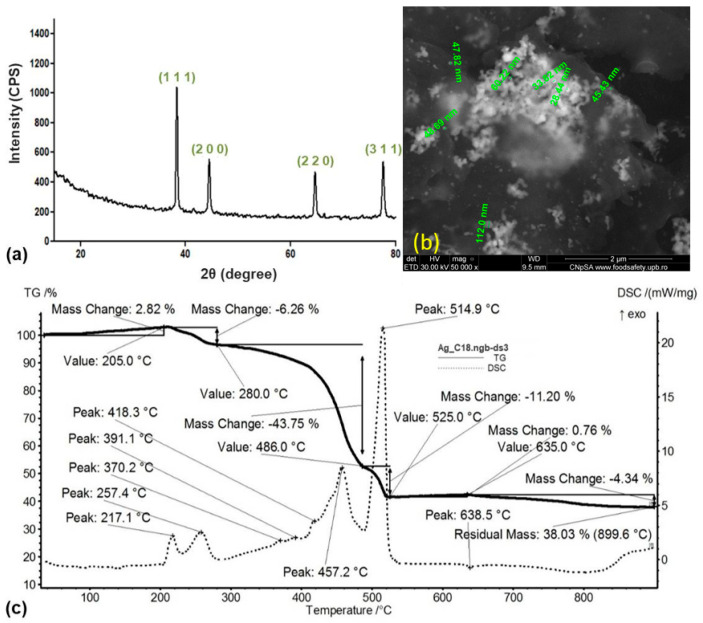
X-ray diffraction pattern (**a**), SEM micrograph (**b**), and TG-DSC results (**c**) of Ag@C_18_ nanoparticles.

The XRD diffractogram (Figure 1a) evidenced the purity of the synthesized powdery sample, with metallic Ag as the only crystalline phase. According to specialized data (PDF no. 00-004-0783) and compliant with previous studies [45,46], diffraction maxima at 2θ values of ~38°, ~44°, ~64°, and ~78° were assigned to the (1 1 1), (2 0 0), (2 2 0), and (3 1 1) crystallographic planes of face-centered cubic crystallized Ag.

The thermal behavior of the metallic powder was evaluated by correlating mass variations and thermal effects recorded by thermogravimetric analysis (TGA) and differential scanning calorimetry (DSC), respectively. The mass variation started with an initial 2.82% mass gain, which occurred below 205 °C due to the surface oxidation of particles (evidenced through the 91.8 °C and 139.3 °C weak endothermic peaks). A mass loss of 6.26% was further evidenced up to 280 °C as a result of incipient thermal oxidation (217 °C and 257 °C exothermic events) of surface-conjugated organics. The most significant mass loss (43.75%) occurred between 280 and 486 °C and was correlated with the thermal degradation of the long-chain stabilizing agent (asymmetrical exothermic event at 457.2 °C accompanied by overlapped secondary oxidations at 370 °C, 391 °C, and 418 °C). The complete thermal degradation of carbon-based residuals occurred between 486 and 525 °C by the most prominent exothermic event (with 514.9 °C maximal outcome, which was correlated with a fast mass loss of 11.2%). The collected derivatogram further evidenced a 0.76% mass gain at 635 °C and a final 4.34% mass loss (up to 900 °C). The residual mass of 38.03%, particularly assigned to the metallic residue, indicated the increased amount of sodium stearate molecules that were conjugated onto the surface of metallic particles.

The SEM results (Figure 1b) evidenced the presence of particle aggregates (bright areas) within an organic matrix (areas of reduced intensity). Aggregates consisted of spherical particles with a mean particle size of ~54 nm.

### 2.2. Physicochemical Investigation of nGO Nanomaterial

A high-purity GO powdery sample resulted from using this protocol, as evidenced through the sharp (0 0 1) diffraction plane (2θ = 10.63°), which confirms the oxidation of graphitic layers [47,48]. The second diffraction maxima, identified at 2θ = 43°, was associated with the short-range stacking of exfoliated graphitic layers [49,50].

**Figure 2:**
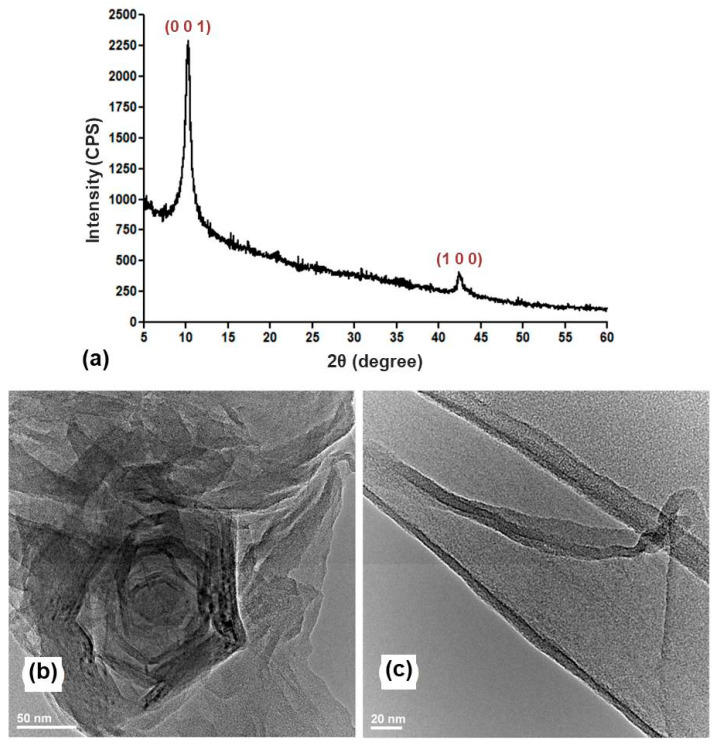
X-ray diffraction pattern (**a**) and (TEM) micrographs (**b**,**c**) of nGO.

These results were supported by TEM analysis (Figure 2b), which evidenced the successful exfoliation of graphitic layers and the formation of GO sheets that adopt a folded arrangement. Upon closer observation, the formation of defect-free ultra-thin sheets was noted (Figure 2c).

### 2.3. Physicochemical Investigation of Ag@C_18_-nGO Coatings

**Figure 3:**
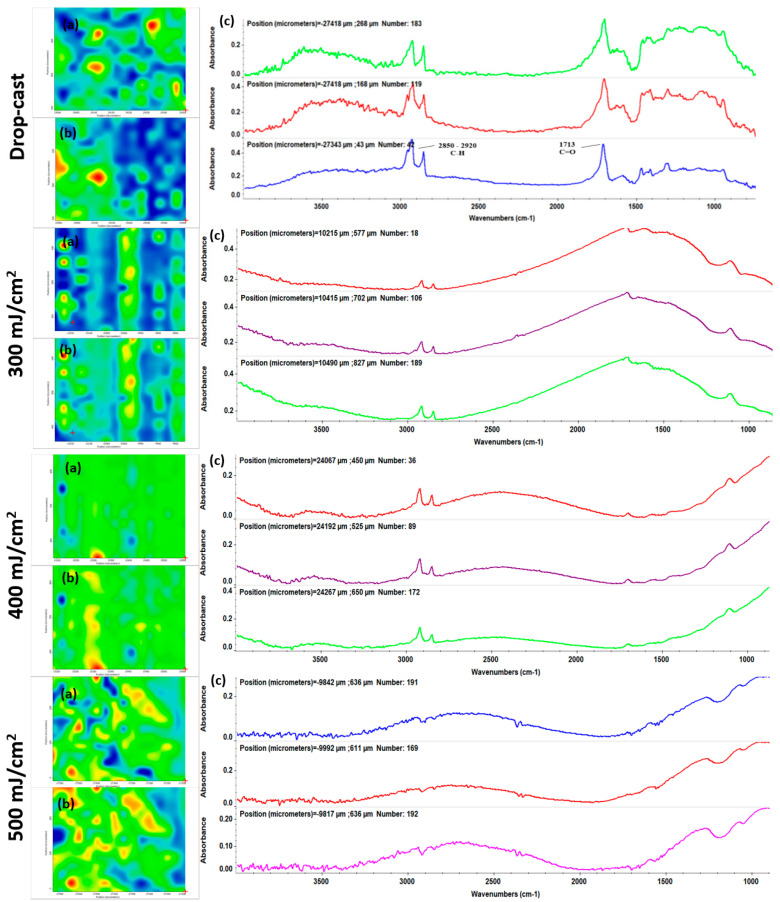
IR maps (**left**) resulting from monitoring the intensity of C–H (set a) and C=O (set b) and corresponding IR spectra (**right**, set c) of Ag@C_18_-nGO coatings obtained at differences laser fluences.

In our experiments, drop-cast (pristine material) and MAPLE-processed (300, 400, 500 mJ/cm^2^) samples were comparatively investigated by IRM analysis to identify the optimal laser fluence for processing the Ag@C_18_-nGO mixture. Complementary IR spectra (set c in Figure 3, resulting from analysis of different points in each sample) and IR maps (a, b sets in Figure 3, corresponding to the absorbance intensity distribution of C–H and C=O moieties, respectively) were collected for all samples (Figure 3).

The IR spectra of the Ag@C_18_-nGO drop-cast sample showed the clear presence of asymmetric (~2920 cm^−1^) and symmetric (~2850 cm^−1^) C–H vibrations originating from the highly abundant –CH_2_– moieties within the stearic acid [51,52]. These maxima were accompanied by weak IR peaks (identified at higher wavenumbers) resulting from C–H vibrations within the terminal methylene of the fatty acid. In addition, –CH_3_ bending was observed at ~1320 cm^−1^ [53,54], supporting the likelihood that stearate-conjugated AgNPs were present within the mixture. Specific GO molecular vibrations were observed at ~1713 cm^−1^ due to the intense stretching of C=O [55,56].

Though reduced in intensity, C–H and C=O molecular vibrations were also present in the IR spectra of MAPLE coatings obtained at 400 mJ/cm^2^ together with the GO-originating C–O stretching (~1110 cm^−1^) [57,58]. These maxima were more reduced and even absent in the case of lower (300 mJ/cm^2^) and higher (500 mJ/cm^2^) laser fluences due to the inefficient or altered laser transfer of Ag@C_18_-nGO composite, respectively. This outcome was further supported by the corresponding IR maps in which color variations are directly related to the intensity of monitored absorbance bands ranging from red (highest) to blue (lowest).

IRM results indicated that the 400 mJ/cm^2^ laser fluence is optimal for the MAPLE processing of Ag@C_18_-nGO coatings, as evidenced by complementary IR spectra (compositional integrity and stoichiometry) and IR maps. We decided to perform additional investigations only on the Ag@C_18_-nGO coatings obtained with the middle laser fluence.

**Figure 4:**
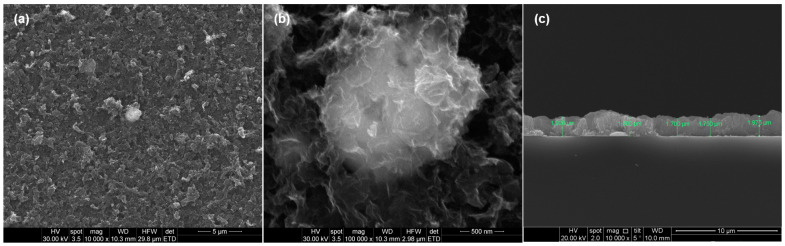
Top-view (**a**–**c**) and cross-section (**c**) SEM micrographs of Ag@C_18_-nGO coatings obtained at 400 mJ/cm^2^ laser fluence.

Consistent with IR maps, the top-view SEM images showed that the Ag@C_18_-nGO composite material processed at 400 mJ/cm^2^ laser fluence continuously and uniformly covered the substrate (Figure 4a), resulting in homogenous coatings with an irregular surface and a rather porous aspect (Figure 4b). As can be seen (Figure 4c), the thickness varies between 1.7 and 2 µm, which delineates the small agglomeration tendency.

This outcome evidenced the preserved morphology and dimension of nGO after MAPLE, in compliance with TEM and IRM results.

### 2.4. In Vitro Biocompatibility Assessment of the Ag@C_18-_nGO Coatings

**Figure 5:**
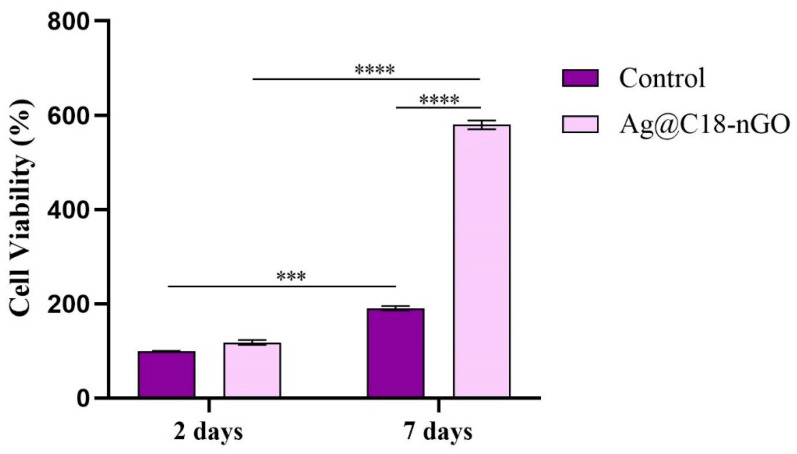
Cell viability and proliferation pattern of MC3T3-E1 cells after 2 and 7 days of contact with the pristine and Ag@C_18_-nGO -coated samples (*** *p* ≤ 0.001; **** *p* ≤ 0.0001).

A colorimetric MTT assay was employed to investigate the potential of Ag@C_18_-nGO coatings to sustain and promote MC3T3-E1 cell adhesion and proliferation (Figure 5). After 2 days of culture, no significant changes in the MC3T3-E1 cellular metabolic activity were triggered by Ag@C_18_-nGO-coated samples as compared with the experimental control. In contrast, after 7 days of culture, MC3T3-E1 cell viability exhibited a statistically significant (*p* ≤ 0.0001) increase in cell metabolic activity in response to Ag@C_18_-nGO coatings compared to non-coated substrates. Moreover, the obtained results revealed that both samples stimulate MC3T3-E1 preosteoblasts’ proliferation, as a statistically significant increase in cell viability was observed when comparing results between the two experimental times. However, the nanostructured coatings notably enhance the proliferative activity of MC3T3-E1 cells, revealing that the engineering strategy provides a more favorable environment for cellular proliferation.

**Figure 6:**
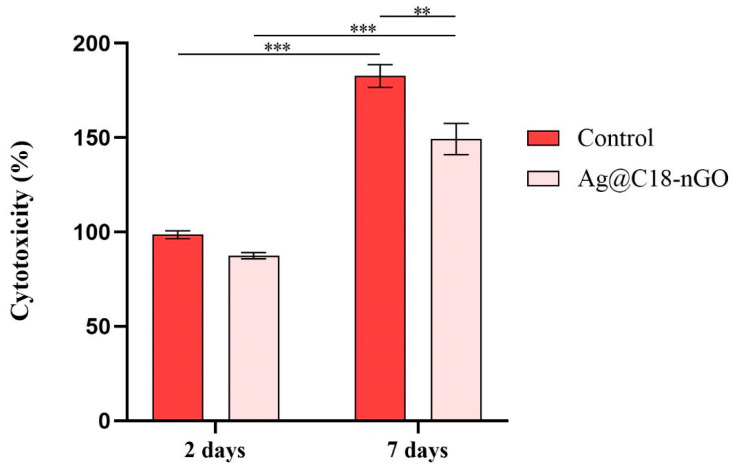
Ag@C_18_-nGO coatings’ cytotoxic potential as revealed by the LDH levels released by MC3T3-E1 cells after 2 days and 7 days of culture. The experimental control is represented by the non-coated surface (** *p* ≤ 0.01; *** *p* ≤ 0.001).

The cytotoxic potential of Ag@C_18_-nGO coatings was assessed by quantifying the amount of LDH released into the culture medium by preosteoblasts damaged as a result of cell–material interaction (Figure 6). After 2 days of culture, the LDH levels were similar in both experimental conditions. Increasing the culture time to 7 days produced a significant increase (*p* ≤ 0.001) in the LDH release in the collected culture media from both samples as compared with culture media samples collected after 2 days. However, the LDH activity was statistically significantly lower in the culture media samples collected from the Ag@C_18_-nGO-coated samples as compared with control samples, showing that in the absence of the nanostructured surface, the murine preosteoblasts are more prone to cellular membrane damage. These results highlight the low cytotoxicity of the activity—statistically significantly lower than Ag@C_18_-nGO coatings, which act as a protective substrate against damage induced by the pristine titanium.

**Figure 7:**
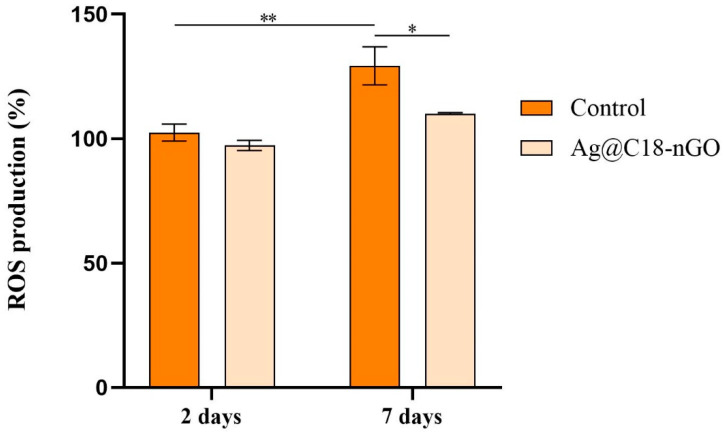
Impact of Ag@C_18_-nGO coatings on ROS production after 2 and 7 days of MC3T3-E1–material interaction as revealed by ROS–Glo H_2_O_2_ assay (* *p* ≤ 0.5; ** *p* ≤ 0.01).

The potential of Ag@C_18_-nGO coatings to induce oxidative stress was assessed by quantifying the levels of H_2_O_2_ released in the culture medium as a measure of ROS production (Figure 7). After 2 days, no differences between H_2_O_2_ released in the culture media were observed between the experimental conditions, showing that Ag@C_18_-nGO coatings do not modify H_2_O_2_ levels. After 7 days of culture, a statistically significant (*p* ≤ 0.1) enhancement in H_2_O_2_ levels was observed in the control sample medium as compared with 2 days of culture, showing that the pristine sample induces ROS generation in time. In contrast, the nanostructured coatings do not induce oxidative stress in murine preosteoblasts, as H_2_O_2_ levels were not augmented by increasing the cell–material interaction period, and H_2_O_2_ levels were significantly lower at this time point (*p* ≤ 0.5) as compared with H_2_O_2_ levels registered in the control.

**Figure 8:**
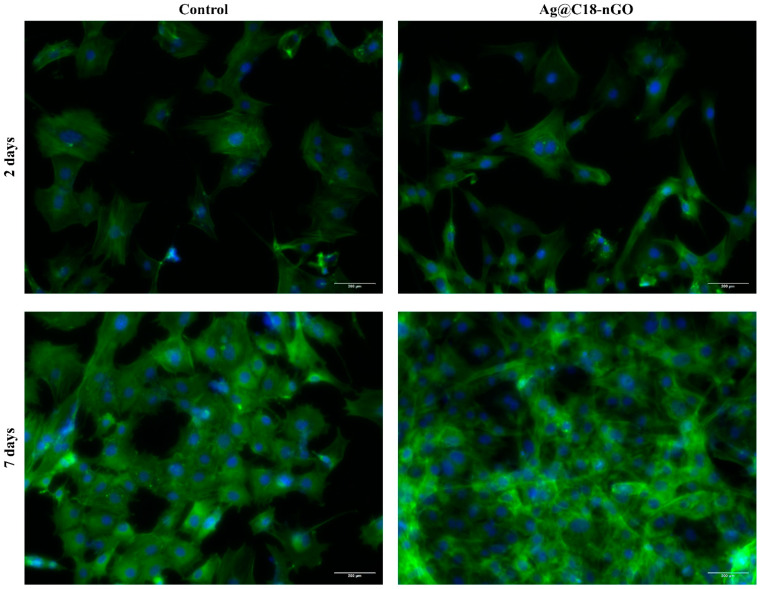
Fluorescence micrographs of MC3T3-E1 cells’ cytoskeleton after 2 and 7 days of culture in contact with pristine and Ag@C_18_-nGO-coated samples after fluorescent staining of actin filaments (green) and cell nuclei (blue) with FITC-phallodin and DAPI (scale bare: 200 μM).

To reveal the cellular morphology of MC3T3-E1 cells cultured in contact with Ag@C_18_-nGO coatings, actin filaments were stained with phalloidin-FITC (Figure 8). After 2 days of culture, MC3T3-E1 attached firmly on both pristine and nanostructured coating, presenting the typical spindle-shaped morphology of preosteoblast cells on both samples. However, prolonging the preosteoblasts–sample contact to 7 days revealed that in the absence of the nanostructured coating, preosteoblasts fail to adopt an elongated morphology characterized by short actin filaments condensed around the nuclei. The nanostructured coatings allowed MC3T3-E1 cells to develop and be distributed across the entire Ag@C_18_-nGO-coated sample surface in a dense cellular network, with preosteoblasts presenting an elongated morphology characterized by a well-developed cytoskeleton.

### 2.5. Microbiological Evaluation of Ag@C_18_ Nanoparticles and Ag@C_18_-nGO Coatings

We investigated the antimicrobial effects induced by synthesized Ag@C_18_ NPs against bacterial strains that undergo important antibiotic resistance modifications [59,60] and possess significant ethological implications in community and nosocomial infections [61,62]. Significant inhibitory effects were induced by Ag@C_18_ NPs against *P. aeruginosa* and *E. faecalis* strains, with effective concentrations of 4 μg/mL and 2 μg/mL, respectively. When compared to highly potent cephalosporin antibiotics, lower Ag@C_18_ concentrations were required for inhibiting the growth of *E. coli* (2 μg/mL Ag@C_18_ vs. 4 μg/mL Cefuroxime or Cefoperazone) and *S. aureus* (1 μg/mL Ag@C_18_ vs. 8 μg/mL Cefuroxime vs. 4 μg/mL Cefoperazone).

Our results revealed the significant antibacterial efficacy of synthesized Ag@C_18_ NPs, which demonstrated more intense inhibitory effects against the Gram-positive strains. Further, the ability of Ag@C_18_-nGO coatings to interfere with the development of monospecific bacterial biofilms was assessed at different time points (24, 48, and 72 h).

**Figure 9:**
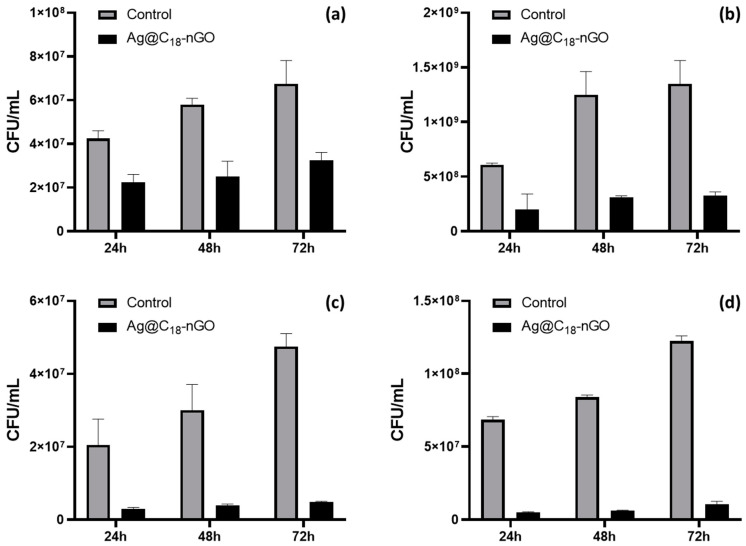
Microbial biofilm development of *E. coli* (**a**), *P. aeruginosa* (**b**), *S. aureus* (**c**), and *E. faecalis* (**d**) after different incubation periods with Ag@C_18_-nGO coatings obtained at 400 mJ/cm^2^; expressed as CFU/mL values.

A reduced but sustained inhibitory effect was observed against Gram-negative bacterial biofilms (Figure 9a,b). Compared to uncoated substrates (pristine), the Ag@C_18_-nGO-coated samples proved more effective during the initial colonization of *E. coli* (days 1 and 2) and late colonization of *P. aeruginosa* (days 2 and 3), with a maximal inhibitory efficiency recorded after 48h regardless of the considered strain.

Similar results were observed in the case of Gram-positive strains (Figure 9c,d), as the Ag@C_18_-nGO-coated samples produced a sustained inhibition of bacterial biofilms with a bacterial population reduction of at least one order of magnitude for both bacteria which was maintained throughout the entire experimental period. These results were in agreement with the intrinsic antibacterial activity of Ag@C_18_ NPs.

## 3. Discussions

Owing to their remarkable and extensive antimicrobial efficacy, reduced antipathogenic resistance, and extended performance against antibiotic-resistant microorganisms, silver nanoparticles (AgNPs) are potent candidates for fabricating new antimicrobial formulations [63,64]. Various methods have been successfully used for obtaining AgNPs with tailored physicochemical properties and an adaptable range of capabilities, including physical, chemical, biological, and physicochemical (employing external energy sources to conduct chemical reactions) approaches [63,65,66,67]. The chemical methods provide an application-related tuning in the chemical reduction processes of silver salts, representing the simplest, most efficient, and easy-to-handle synthesis strategies for AgNPs [66,67,68,69].

Herein, AgNPs were synthesized in the presence of D-glucose and sodium stearate, which represent the reducing and stabilizing agents, respectively. The fast color changes that occurred by gradually adding the metallic precursor solution within the organic solution were an early indicator of the formation of Ag@C_18_ nanoparticles, as this process is correlated with the reduction of Ag^+^ to Ag^0^ [70,71]. The wet chemical synthesis protocol enabled the easy and fast formation of high-purity Ag@C_18_ nanoparticles with face-centered cubic crystalline lattice (Figure 1a), sphere-like morphology, and ~54 nm mean particle size (Figure 1b). In addition, the thermal analysis revealed an increased amount of sodium stearate molecules (>60%) conjugated on the surface of nanosilver particles (Figure 1c). In terms of microstructure, and given the selection of a mild reducing agent and a long-chain stabilizing agent, our finding are consistent with previous studies regarding the fabrication of fatty acid-capped (@C_18_) nanosilver [72,73].

Graphene oxide, a versatile two-dimensional nanomaterial with an extensive surface area, outstanding electrical, mechanical, and thermal behavior, and intrinsic biocompatibility and antimicrobial activity [39,74,75,76,77], has been highly explored for modern biomedical applications, including biosensing and bioimaging, drug delivery platforms, and tissue engineering scaffolds [78,79].

In our study, a modified multi-step Hummers’ protocol was applied for obtaining the GO by using a H_2_SO_4_/K_2_S_2_O_8_/P_2_O_5_ mixture for the thermal oxidation of graphitic layers, H_2_SO_4_/KMnO_4_ for intercalated oxidation, H_2_O_2_ for maximized oxidation and chemical exfoliation, and HCl for additional purification. The formation of defect-free ultra-thin GO sheets that adopted a particular folded arrangement (Figure 2b,c) was correlated with the successful interlayer intercalation of oxygen-containing functions and water molecules. This outcome complies with previous studies on obtaining highly folded GO sheets with wrinkled nanosheet surfaces using modified Hummers’ methods [80,81].

Highly biocompatible nanostructured coatings with application-related biofunctional outcomes (immunomodulation, antimicrobial efficiency, antitumor activity) have been developed through the MAPLE technique [82,83], which represents an attractive and tunable strategy for the unaltered, stoichiometric, and stable transfer of small-molecule or macromolecule organics. With the aim to achieve the unaltered and efficient transfer of MAPLE-processed coatings, the optimal selection of laser parameters (mainly, the laser energy distribution over the effective target area) is generally required. Mostly, compositional and microstructural tuning studies between preprocessed materials and materials processed at different laser fluences are considered [84,85]. The IRM results, consisting in complementary IR spectra (Figure 3c) and IR maps (Figure 3a,b) of drop-cast (pristine) and MAPLE samples revealed that the 400 mJ/cm^2^ laser fluence is optimal for the MAPLE processing of Ag@C_18_-nGO coatings. Thus, uniform nanostructured coatings with an irregular surface and a rather porous aspect were obtained (Figure 4). In terms of microstructure and thickness (<2 μm), similar results were reported for GO/Ag coatings obtained by electrophoretic deposition, which is preferred for the improvement of metallic surfaces due to its industrial use [86,87,88].

To evaluate the biological profile of MAPLE-processed nanostructured coatings, MC3T3-E1 murine preosteoblasts were used during complementary cellular assays. A statistically significant increase in the cell viability and proliferation ability (Figure 5), together with a significant cytotoxicity decrease (Figure 6), was demonstrated by preosteoblasts after they were cultured in the presence of Ag@C_18_-nGO-coated samples when compared to uncoated titanium substrates. Furthermore, the nanostructured coatings did not induce oxidative stress in murine osteoprogenitor cells, as H_2_O_2_ levels registered a significant decrease after both incubation periods (2 and 7 days, Figure 7), while the normal development, distribution, and growth of MC3T3-E1 cells was sustained (Figure 8).

Previous studies reported the high biocompatibility of AgNPs-decorated GO obtained by laser ablation with respect to normal osteoblasts [89] and reported the improved bioactivity and osteogenic ability of implantable metallic surfaces modified with AgNPs/GO-layered titania oxide nanotube array [90,91] or polyester coating [92]. Taken together, from all the assays performed to assess the biological performance of Ag@C_18_-nGO coatings, we can conclude that the coating strategy enhances the cell–substrate interaction of MC3T3-E1 cells, promoting and supporting cell adhesion, viability, and proliferation. These results have a significant impact for the potential of the novel nanostructured coating in future biomedical applications.

As AgNPs possess strong and effective action against a wide range of planktonic, sessile, antibiotic-sensitive, and antibiotic-resistant bacteria, using nanosilver represents an attractive and promising strategy for developing new and efficient antimicrobials. For the excellent intrinsic antibacterial action of AgNPs, the following mechanisms have been proposed: (i) destabilization and piercing of bacterial membrane, due to the nanosize-related reactivity for structural macromolecules containing phosphorous and sulfur [93,94]; (ii) inactivation and denaturation of vital macromolecules, due to nanosize-related reactivity and electrostatic interactions mediated by the released Ag^+^ ions [95,96]; and (iii) impaired cellular respiratory chain and/or signal-transduction pathways due to the Ag^+^-mediated oxidative stress [97,98].

AgNPs are highly potent antimicrobials; they are less toxic than antibiotic formulations [64,65,69,99,100,101] and are also effective in reducing microbial attachment and biofilm formation. As a result, there is increased interest in their potential application in fabricating coatings for performance-enhanced medical devices [22,26,31,65,102]. The anti-infective efficiency of nanosilver-based biomaterials has been reported against a broad spectrum of clinically relevant pathogens, including *Bacillus subtilis*, *Enterococcus faecalis*, *Klebsiella pneumoniae*, *Pseudomonas aeruginosa*, *Salmonella enterica*, *Staphylococcus epidermidis*, *Streptococcus mutans*, and *Streptococcus pyogenes* [38,63,68,103,104,105,106,107,108,109,110,111,112,113,114,115,116,117,118]. We evidenced the intrinsic antibacterial effects of Ag@C_18_ NPs against *P. aeruginosa* and *E. faecalis* strains, as it demonstrated effective inhibitory concentrations comparable with others reported for biosynthesized AgNPs [109,118]. AgNPs could act differently on Gram-negative and Gram-positive bacteria, as one of their targets is the cellular wall. AgNPs adhere to microbial cell surface and produce membrane damage and altered transport activity; this interaction could be influenced by the thickness of the peptidoglycan layer—which differs among Gram-negative and Gram-positive cells—and by the electric charge of the cellular surface [119]. Another proposed antibacterial mechanism is that AgNPs penetrate inside microbial cells and interact with cellular components such as nucleic acids, proteins, and lipids, impairing the functionality of the cells. Moreover, AgNPs cause an increased production of ROS inside the microbial cells, leading to cell damage. Depending on the amount and properties of silver NPs, their presence in the bacterial cell could lead to effects ranging from mild effects, such as interference with the cellular signaling system, up to irreversible cell damage ultimately causing their death [120].

While green-synthesized and polysaccharide-capped AgNPs showed a slightly reduced antibacterial activity against *E. coli* and *S. aureus* when compared to commercial antibiotics [115,121], the developed Ag@C_18_ NPs exhibited superior inhibitory action against selected bacteria, with much lower inhibitory concentrations than reference antibiotics.

Herein, we reported the important antibacterial efficacy of synthesized Ag@C_18_ NPs against bacterial strains that undergo notable antibiotic resistance modifications [59,60] and possess significant ethological implications in community and nosocomial infections [61,62] with more intense inhibitory effects against Gram-positive strains.

This outcome relies on the compositional and microstructural differences between selected bacteria. Though the cell wall of Gram-negative bacteria consists of a much thinner and more AgNP-susceptible peptidoglycan layer [94,122], the lipopolysaccharide outer membrane provides additional hydrophilic protection against nonpolar moieties, as in the case of the long-chain fatty acid that is abundantly conjugated on the surface of nanosilver. On the other hand, the much thicker and porous peptidoglycan layer within Gram-positive bacteria, together with the constituent teichoic acids (which facilitate electrostatic interactions with the conjugated fatty acid), may accelerate the oxidative damage caused by the intracellular release of metallic ions and accelerated generation of reactive species [123,124].

In addition to their excellent biocompatibility and antipathogenic efficacy, the fine-tuned modulatory effects of silver-based nanomaterials on reparative and regenerative processes validate their use as multipurpose platforms for the infection-free integration of orthopedic implants. For instance, the use of AgNPs as doping agents in bone cements based on calcium phosphate [125] and resin [126] has been reported as a promising strategy for the management of periprosthetic infections caused by relevant Gram-negative and Gram-positive strains. The sole modification of titanium surfaces [127,128] and biodegradable polyester scaffolds [129] with AgNPs has been validated for the fabrication of osteogenic and anti-infective orthopedic devices. In comparison with bare titanium-based devices, implants coated with AgNP-loaded organosilica-based hybrid materials [130] and wires modified with silver multilayer coatings [131] reduced post-implantation inflammation and led to enhanced bone healing during ligament reconstruction surgery in animal models.

Preventing or limiting post-implantation infections has been also evidenced in the case of titanium-based devices modified with hydrothermally transformed [132] or plasma-sprayed [133] hydroxyapatite coatings enriched with nanosilver: additional enhanced bone stability and osteoinductive ability has been reported. Embedding nanosilver within polydopamine-based coatings [134] and hierarchical layers [135] proved an effective strategy to boost the infection-free osseointegration of thermoplastic implantable devices, as evidenced against *E. coli* and *S. aureus* strains, with additional osteogenic and angiogenic effects.

In addition to their intrinsic antimicrobial efficacy, AgNPs have demonstrated outstanding synergistic activity when associated with various natural or synthetic compounds [103] or carbonaceous nanomaterials (such as graphene and graphene oxide) [39]. Therefore, we further evaluated the ability of Ag@C_18_-nGO coatings to interfere with the development of monospecific bacterial biofilms at different time points (24, 48, and 72 h).

In the case of Gram-negative bacteria, the Ag@C_18_-nGO-coated samples produced a maximal inhibitory efficiency after 48 h regardless of the considered strain (Figure 9a,b) which may be possible by extending previous idea regarding the hydrophilic barrier role of the bacterial cell wall. By contrast, a more effective and time-sustained biofilm inhibition ability of Ag@C_18_-nGO coatings was evidenced against Gram-positive strains (Figure 9c,d), which is consistent with previous data regarding the antibacterial efficiency of Ag@C_18_ NPs.

As evidenced for titanium and thermoplastic implants, coating their surface with AgNPs/antibiotic-loaded natural protein layers [136] or AgNP-loaded polysaccharide/protein scaffolds reinforced with hydroxyapatite nanoparticles [137] resulted in enhanced joint biomechanics and improved osseointegration (by promoting new bone formation while inhibiting bone remodeling and resorption). Such materials have been successfully proposed for modulating the osseointegration process following ligament reconstruction surgery. In addition to boosting the bioactivity and enhancing the biomechanics of carbonaceous coatings deposited onto titanium-based implants [86,138], our results are in agreement with other studies regarding the anti-infective efficiency of GO/Ag nanostructured coatings [92,139].

## 4. Materials and Methods

### 4.1. Materials

To synthesize nanostructured materials, necessary analytical-graded chemicals were purchased from Sigma-Aldrich (Merck Group, Darmstadt, Germany) and used throughout our experiments with no additional purification. They included the following: silver nitrate (AgNO_3_), D-glucose (C_6_H_12_O_6_), sodium stearate (C_18_H_35_NaO_2_), sodium hydroxide (NaOH), sodium chloride (NaCl), sulfuric acid (H_2_SO_4_), potassium persulfate (K_2_S_2_O_8_), phosphorous pentoxide (P_2_O_5_), potassium permanganate (KMnO_4_), hydrogen peroxide (H_2_O_2_), hydrochloric acid (HCl), acetone (C_3_H_6_O), ethanol (C_3_H_6_O), and dimethyl sulfoxide (DMSO). Most reagents involved in the biological and microbiological assays were also provided by Sigma-Aldrich (Merck Group, Darmstadt, Germany).

### 4.2. Synthesis Methods

#### 4.2.1. Synthesis of Silver Nanoparticles (Ag@C_18_)

In agreement with previous studies, an easy and high-yield reduction protocol was used for the synthesis of nanosilver [31,140]. Briefly, the metallic precursor solution, AgNO_3_1%, was added dropwise to the reducing/stabilizing solution (C_6_H_12_O_6_ and C_18_H_35_NaO_2_ dispersed in NaOH 10% solution). Once the reaction was completed under continuous stirring and the suspension stabilized, NaCl was added; then, the resulting slurry was collected, washed, and air-dried; finally, a powdery sample was obtained.

#### 4.2.2. Synthesis of Graphene Oxide Nanosheets (nGOs)

A modified Hummers’ protocol was used for the synthesis of nGO [48,54,141]. Briefly, pre-oxidized graphite (obtained by the stirring-assisted dispersion of graphite powder in H_2_SO_4_/K_2_S_2_O_8_/P_2_O_5_ mixture followed by multiple steps of washing, filtration, and drying) was dispersed in concentrated H_2_SO_4_ solution, with the addition of KMnO_4_, at a temperature close to 0 °C. The mixture was subsequently stirred at 35 °C and 80 °C (after deionized water dilution) for 2 h at each step, washed, and redispersed in 30% H_2_O_2_ solution; then, the resulting precipitate was filtered, washed with 3% HCl solution and deionized water (until it reached neutral pH), and, finally, air-dried at 60 °C for 24 h.

#### 4.2.3. MAPLE Processing of Ag@C_18_-nGO Coatings

The MAPLE experiment used a stainless-steel ultra-high vacuum reaction chamber utilizing a KrF* excimer laser source (λ = 248 nm, τ_FWHM_ ≤ 25 ns) running at a repetition rate of 15 Hz. Following its freezing, using liquid nitrogen, the solution containing 2.5% Ag@C_18_-nGO (1:4 wt%) in DMSO was transferred in copper holders; thus, solid targets were obtained. The laser beam was directed at a 45° angle with respect to the target surface. The ablated material was collected either onto titanium (Ti) (medical-grade purity) of 12 mm diameter or on flat, square, commercially available Si (1 0 0) substrates of 15 × 15 mm^2^ that were placed 3.5 cm apart in a parallel direction to the targets.

All depositions were performed at 0.1 Pa with a laser spot size of ~30 mm^2^ and 18,000 pulses at laser fluences of 300, 400, and 500 mJ/cm^2^.

### 4.3. Physicochemical Characterization

#### 4.3.1. X-ray Diffraction (XRD)

To retrieve information on the purity and crystallinity of the obtained powdery samples, they were analyzed using the Cu_Kα_ radiation (λ = 1.056 Å) of an XRD-6000 Shimadzu diffractometer (Duisburg, Germany). Scans were collected using Bragg–Brentano geometry, with 0.5° incidence angle in 15–80° and 5–60° diffraction angle ranges.

#### 4.3.2. Thermal Analysis

Thermal analysis of the metallic powder was performed under a normal atmosphere using STA 449C Jupiter equipment from Netzsch (Selb, Germany). For this investigation, a small amount of powder was introduced in an alumina crucible and heated, starting from room temperature (RT) up to 1000 °C, with a 1 °C/min heating rate and 50 mL/min airflow.

#### 4.3.3. Transmission Electron Microscopy (TEM)

TEM investigation of the carbonaceous sample was performed in transmission mode with a Tecnai^TM^ G2 F30 S-TWIN high-resolution electron microscope (Thermo Fischer Scientific, Hillsboro, OR, USA). Before analysis, a small amount of powder was dispersed in ethanol under sonication, placed on the carbon-coated copper grid, and RT-dried.

#### 4.3.4. Scanning Electron Microscopy (SEM)

The microstructure of metallic powder and laser-processed coatings was investigated using an FEI Inspect S electron microscope (Thermo Fischer Scientific, Hillsboro, OR, USA). Small powder amounts and MAPLE-modified substrates were fixed, with conductive tapes, onto aluminum holders and then analyzed with secondary electron beams.

#### 4.3.5. Infrared Microscopy (IRM)

A Nicolet iN10 MX FT-IR microscope (Thermo Fischer Scientific, Waltham, MA, USA) was employed to collect complementary infrared (IR) spectra and maps. For each coating batch, 32 measurements were recorded in transmission mode (4000–750 cm^−1^ wavenumber range, with 4 cm^−1^ resolution) and then co-added and converted to absorbance with the OmincPicta 8.0 software (Thermo Fischer Scientific).

### 4.4. Biological Evaluation

To investigate the biocompatibility of Ag@C_18_-nGO-coated samples, the mouse preosteoblasts’ MC3T3-E1 cell line (CRL-2593, ATCC) was selected as an in vitro cellular model. Before each experiment, uncoated substrates (pristine titanium) and Ag@C_18_-nGO-coated substrates were sterilized prior to cell seeding by UV light exposure and placed in 24-well sterile culture plates. For all experiments, cells were seeded at an initial density of 1 × 10^4^ cells/cm^2^ on the sample’s surface as 20 μL/sample droplet positioned in the center of each sample. After 1h, all samples were immersed in Dulbecco’s Modified Eagle Medium (DMEM), to which were priorly added 10% FBS and 1% penicillin/streptomycin mixture (10,000 units/mL penicillin and 10 mg/mL streptomycin). The resulting bioconstructs were maintained in culture for 7 days; the culture media was refreshed every other day. The pristine titanium substrates were processed identically to the nanostructured samples for all biological assays and will be referred to as controls.

To reveal the impact of Ag@C_18_-nGO coatings on MC3T3-E1 cell viability, MTT assay was performed using 3-(4,5-dimethilthiazol-2-il)-2,5-dipheniltetrazolium bromide (MTT) reduction reagent. Briefly, following 2 and 7 days of cell–material interaction, the culture medium was discarded and replaced with a fresh solution of MTT (1 mg/mL). After 4 h of incubation at 37 °C, the resulting formazan crystals formed on the material surfaces were solubilized in 2-propanol, and the absorbance of the solution was read at 550 nm using the FlexStation III multimodal reader (Molecular Devices, San Jose, CA, USA). The results were obtained as a % of cell viability considering the mean OD from the experimental control 48 h post-seeding as 100% cell viability.

To investigate the cytotoxic potential of the nanostructured coatings, culture media samples were harvested at 2 and 7 days of cell–material interaction and further used for the spectrophotometric evaluation of lactate dehydrogenase (LDH) activity. For this purpose, the harvested culture medium was mixed with the components of the TOX-7 kit (LDH-based in vitro toxicology assay kit) according to the manufacturer’s indications. After being incubated at room temperature in the dark for 30 min, the absorbance of the samples at 490 nm was verified using the FlexStation III multimodal reader. The results were obtained as % of LDH release considering the mean OD from experimental control 48 h post-seeding as 100% cytotoxicity.

The effect of Ag@C_18_-nGO-coated samples on reactive oxygen species (ROS) production was investigated by ROS–Glo H_2_O_2_ assay (Promega, Madison, WI, USA), which quantifies the level of hydrogen peroxide (H_2_O_2_) released in the culture medium as a measure of oxidative stress. At each time point, before 6h of fulfilling the 2 and 7 days of cell–material interaction, H_2_O_2_ substrate was added at a final concentration of 25 μM and incubated at 37 °C in a humidified atmosphere of 5% CO_2_. At the end of the 6 h, 50 μL of media from each sample was collected and further mixed with 50 μL of ROS–Glo Detection Solution in an opaque 96-well plate. After 20 min of incubation at room temperature, the luminescence of each well was determined using the FlexStation III multimodal reader. The results were obtained as % of ROS release considering the mean OD from experimental control 48 h post-seeding as 100% ROS production.

The cellular morphology of MC3T3-E1 preosteoblasts cultured on nanostructured coatings was investigated by fluorescent staining of cytoskeleton actin filaments. With this in mind, the culture media was discarded from each sample and replaced with a 4% paraformaldehyde solution for cell fixation. After 20 min, samples were washed with PBS, and cells were further permeabilized for 1 h using 0.1% Triton X-100/2% bovine serum albumin solution. For fluorescent labeling of actin filaments and cell nuclei, samples were stained with fluorescein isothiocyanate (FITC)-conjugated phalloidin at 37 °C and with 4, 6-diamidino-2-phenylindole (DAPI) for 1 h and 20 min, respectively. Fluorescence images were captured with the Olympus IX73 fluorescent microscope equipped with Cell Sense F software V 8.0.2 and were further analyzed with ImageJ V 1.53.

All the assays were performed in triplicate. The results represent the mean of three independent experiments (*n* = 3). GraphPad software 5.03 was employed for data analysis (two-way ANOVA, Bonferroni test). All the presented data represent mean ± standard error of the mean. A *p*-value of ≤0.05 was considered statistically significant.

### 4.5. Microbiological Evaluation

Bacterial strains of *E. coli* (ATCC^®^ 25922), *P. aeruginosa* (ATCC^®^ 27853), *S. aureus* (ATCC^®^ 29213), and *E. faecalis* (ATCC^®^ 29212) were obtained from the American Type Culture Collection (ATCC, Manassas, VA, USA). Antimicrobial tests were performed after the UV sterilization of all coated samples.

#### 4.5.1. Antibacterial Efficacy

A microdilution protocol was applied to determine the minimum inhibitory concentration (MIC) of the synthesized nanosilver. Briefly, binary serial dilutions from the nanopowder were prepared in Luria–Bertani (LB) broth (Thermo Fischer Scientific) using sterile 96-well Nunc plates (Thermo Fischer Scientific) and then inoculated with microbial suspensions of 10^6^ (colony-forming units) CFU/mL (in phosphate-buffered saline (PBS), Sigma/Merck). After standard incubation for 24 h, the MIC values were macroscopically evaluated (through naked-eye analysis) and spectrophotometrically determined (as the lowest concentration of AgNPs that inhibited microbial growth) [142,143].

#### 4.5.2. Antibiofilm Potential

To evaluate the inhibitory efficiency of coatings on bacterial biofilm development, specimens were placed in sterile 24-well Nunc plates containing nutritive broth and then inoculated with microbial suspensions of 0.5 McFarland standard density (1.5 × 10^8^ CFU/mL). After 24 h of incubation, samples were washed with sterile PBS and transferred to new sterile plates containing fresh nutritive broth. After standard incubation for different durations, samples were gently washed and transferred to PBS-containing centrifuge tubes for biofilm detachment and biofilm-embedded bacteria isolation. Final microbial suspensions obtained by serial tenfold dilutions were seeded on LB agar plates (Thermo Fischer Scientific) for viable cell count assay [144,145].

## 5. Conclusions

As interference fixation devices can face microbial contamination and colonization, which may lead to infection occurrence with serious complications, envisioning upgraded prevention and treatment strategies is of vital importance. In this regard, nanosized and nanostructured formulations have been proposed as promising approaches to prevent microbial adhesion or to destroy pathogens following their attachment. Silver-based and carbon-based nanomaterials exhibit remarkable antimicrobial properties that have attracted considerable interest for their potential use in the development of potent anti-infective formulations.

In this context, we have fabricated stearate-conjugated silver (Ag@C_18_) nanoparticles and graphene oxide nanosheets (nGOs) to create Ag@C_18_-nGO coatings suitable for the local modulation of biofilm-associated periprosthetic infections. Both synthesized nanomaterials presented high purity as demonstrated by XRD analysis. SEM and TEM investigations further allowed the morpho-structural characterization of the obtained materials. The Ag@C_18_ NPs displayed spherical morphology and ~54 nm mean size, whereas GO formed defect-free ultra-thin nanosheets that adopted a folded arrangement.

Further, MAPLE processing was chosen for coating deposition with a laser fluence of 400 mJ/cm^2^ being considered optimal. The nanostructured coatings were proven to be highly biocompatible, promoting and supporting cell adhesion, viability, and proliferation of osteoprogenitor cells. Moreover, microbiological data demonstrated sustained antibiofilm activity against Gram-negative and Gram-positive bacterial strains, proving more effective against the latter category. Nonetheless, compared to control substrates, the Ag@C_18_-nGO-coated samples were noted to be more potent during the initial colonization of *E. coli* and late colonization of *P. aeruginosa*, with a maximal inhibitory efficiency recorded after 48 h.

To conclude, the developed Ag@C_18_-nGO coatings exhibited desirable physicochemical and biological properties, holding promise for their further use in modulating the microbial susceptibility of fixation devices used in knee surgery.

## Data Availability

Data are available from authors upon request.
